# Unraveling the Physicochemical Landscape of Organoselenium Compounds in Nanocarrier Systems

**DOI:** 10.1002/tcr.70146

**Published:** 2026-04-17

**Authors:** Romelly Eugenia Rojas Ramírez, Tielle Moraes de Almeida, Daiani Canabarro Leite, Gilson Zeni

**Affiliations:** ^1^ Department of Biochemistry and Molecular Biology UFSM Santa Maria Brazil; ^2^ Department of Physics UFSM Santa Maria Brazil

**Keywords:** forces, nanocarriers, organoselenium, solubility, stabilization

## Abstract

In recent years, publications on organoselenium compounds have increased markedly, expanding their applications in therapeutic, nutraceutical, agricultural, and environmental fields. However, intrinsic properties such as high lipophilicity and chemical reactivity often limit their bioavailability and therapeutic performance. Advances in self‐assembled nanostructures have enabled more efficient delivery, improving solubility and bioavailability while reducing toxicity. This review is the first to focus specifically on the physicochemical interactions governing the stability and performance of organoselenium‐based nanostructures. We analyze recent formulations, emphasizing hydrophobic, van der Waals, electrostatic, and hydrogen bonding interactions that control nanocarrier behavior. Additionally, we discuss emerging strategies and future applications for the rational design of safer and more effective organoselenium delivery systems.

## Graphical abstract

Main nanocarriers employed to enhance the bioavailability and transport of organoselenium compounds. The image illustrates the key interaction forces that stabilize these organoselenium species in solution, enabling their application in drug delivery systems, catalysis, photoelectronic devices, and sensing technologies.

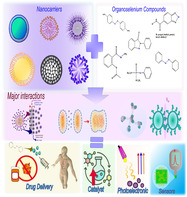

## 1 Introduction

Organoselenium compounds represent a significant class of organochalcogens, offering versatility across various areas of chemistry, including catalysis, organic synthesis, the synthesis of natural products, and the development of new pharmacological compounds. Various studies have verified the pharmacology and toxicology of organoselenium compounds, emphasizing their anti‐inflammatory, hypoglycemic, chemotherapeutic, and antimicrobial activities [1, 2]. However, their clinical translation and technological applications often require carrier systems to enhance their stability, bioavailability, and functionality, since these compounds are usually poorly water‐soluble.

**FIGURE 1:**
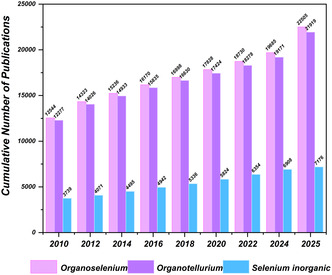
Data extracted from the Web of Science database (November 2025). The dataset includes cumulative publications from 1975, beginning with the period 2010–2025.

The considerable progress in the pharmacology and toxicology of chalcogens has fueled interest in the synthesis and reactivity of new organic and inorganic derivatives [3, 4, 5, 6, 7]. The distinctive reactivity of these compounds has driven extensive research in both areas. A search conducted in the Web of Science database in November 2025 using the terms ‘organoselenium,’ ‘organotellurium,’ and ‘selenium inorganic’ retrieved 22,505; 21,919; and 7176 articles, respectively, highlighting the rapid scientific advancement in this field (Figure 1).

**FIGURE 2:**
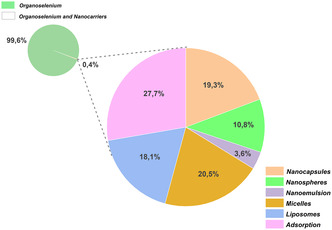
Percentage of the literature found on nanocarriers and organoselenium compounds. Data extracted from the Web of Science database (November 2025). The dataset includes publications indexed between 1975 and 2025.

Recent advancements in nanotechnology, particularly in nanoencapsulation, have significantly expanded the possibilities for delivering active pharmaceutical compounds in aqueous and nonaqueous environments [8, 9, 10] (Figure 2). These developments have been crucial in improving the solubility, bioavailability, dosing, and stability of organoselenium compounds. Innovations in nanostructured drug delivery have led to the design of novel systems that overcome the solubility and stability challenges associated with these compounds [11, 12, 13, 14].

Recent reviews [11, 15, 16] highlight the diversity of self‐assembled nanostructures developed for this purpose, including liposomes, nanocapsules, nanospheres, nanoemulsions, micelles, and polymeric micelles. These delivery systems enhance the therapeutic potential of organoselenium compounds by facilitating their solubility, stability, controlled release, and vectorization in biological environments.

Despite the growing number of studies on organoselenium‐based delivery systems, most reports focus primarily on pharmacological effects. At the same time, fundamental aspects of the physical‐chemical interactions between the nanocarrier components and organoselenium molecules remain underexplored. Understanding these molecular‐level interactions is key to rationally designing nanocarriers with improved performance, including fine‐tuned release mechanisms, physicochemical compatibility, and structural stability under physiological conditions. Moreover, the intrinsic chemical properties of selenium, such as polarizability and unique redox behavior, can play a critical role in the dynamics and architecture of nanostructured systems.

As an emerging field closely linked to the chemistry of organoselenium compounds, this review explores interactions within nanostructured systems and the mechanisms underlying their stability and consequent bioavailability. A detailed understanding of these interactions is essential for the rational design of controlled release systems, providing insights into functionality, performance, and therapeutic efficacy. Recent studies increasingly emphasize the balance between bioavailability and toxicity, highlighting the importance of optimizing nanostructured carriers to enhance absorption while minimizing adverse effects. Elucidating the physicochemical determinants that govern both delivery efficiency and safety is therefore critical for advancing these systems toward practical biomedical applications.

## 2 An Overview of Organoselenium Compounds

### 2.1 Importance of Selenium

Selenium is an essential trace element for human health, playing a crucial role in antioxidant defense, thyroid hormone metabolism, and immune function. Its biological activity is mainly associated with its incorporation into selenoproteins, such as glutathione peroxidases and thioredoxin reductases, which regulate redox homeostasis and protect cells against oxidative stress [17, 18]. Selenium deficiency has been linked to cardiovascular diseases, thyroid dysfunction, impaired immunity, and neurological disorders [18, 19, 20, 21].

In addition to its physiological importance, selenium also has significant implications for cancer prevention. Several studies have demonstrated that adequate selenium intake is associated with a reduced incidence of certain types of cancers, due to its ability to modulate DNA repair mechanisms, apoptosis, and tumor cell proliferation [22]. However, these benefits depend strongly on dose and chemical form, as selenium exhibits a narrow safety margin, being both essential and potentially toxic. Recent studies report a U‐shaped correlation between selenium status and cancer risk. A safe intake has been estimated to range from 110.8 to 124.4 µg/day. A similar U‐shaped association was also observed across multiple subgroups, including men and women, ever‐ and never‐smokers, individuals with BMI < 23 kg/m^2^ and ≥ 23 kg/m^2^, ever‐ and never‐drinkers, and for cancers of the stomach, colon, rectum, and lung [23].

Selenium supplementation has also been investigated in relation to infectious diseases. Recent research highlights its role in improving the immune response to viral infections, including HIV and influenza, by supporting immune cell proliferation and reducing viral replication [24]. These findings reinforce selenium's potential as an adjuvant in nutritional strategies to strengthen immunity. Moreover, selenium has also been investigated in environmental and technological contexts. Its application in nanotechnology, for instance, has enabled the development of selenium nanoparticles (SeNPs) with potential antimicrobial, anticancer, anti‐Alzheimer's, antidiabetic, antioxidant, and antirheumatoid arthritis activities, thereby opening new perspectives for biomedical applications [15, 25]. Therefore, selenium represents a key element at the interface of biology, medicine, and nanotechnology, with significant implications for human health and technological innovation.

### 2.2 Organoselenium Compounds

In recent years, organochalcogen compounds, particularly those derived from selenium and tellurium, have gained increasing attention due to their high reactivity, selectivity in novel reactions, and fundamental roles in pharmacology and toxicology [2, 3, 4, 26]. Studies have demonstrated that organochalcogens facilitate the oxidation of sulfhydryl groups in biologically active molecules [4, 27, 28]. Notably, the neurotoxic effects induced by these compounds in experimental animals have been attributed, at least in part, to their ability to modulate the sulfhydryl groups of glutamatergic neuron receptors [4, 28]. Furthermore, organochalcogen compounds have been shown to inhibit the activity of sulfhydryl‐dependent enzymes, including δ‐aminolevulinate dehydratase (δ‐ALA‐D), Na^+^/K^+^‐ATPase, and 5‐lipoxygenase, likely due to their interactions with sulfhydryl groups [4, 28, 29].

Organoselenium compounds are gaining prominence due to their versatility across multiple disciplines, including biomedicine, materials science, and electronics [1, 5, 6, 30, 31, 32, 33]. These compounds exhibit antibacterial, anti‐inflammatory, and antioxidant properties and hold significant potential in cancer therapy [2, 31, 34]. In materials science, organoselenium compounds are being explored as innovative fluorescent probes, while selenium‐containing heterocycles play a crucial role in the development of organic conductors, semiconductors, and optoelectronic devices [35, 36, 37, 38]. Additionally, advances in nanotechnology have further expanded their biological applications, reinforcing their multidisciplinary importance.

The continuous development of new synthesis routes has enabled the production of a wide range of selenium‐containing molecules with potential applications. Notable examples include additions of selenium to alkenes, alkynyl and vinyl selenides, selenoesters, selenium‐substituted heterocycles, and other complex structures such as indoles, imidazoles, azaindoles, lactams, oxazolines, oxazoles, selenium‐containing carbohydrates, dialkyl(aryl) diselenides, chalcogenophosphates, and others [1]. Selenium serves as an isosteric substitute for sulfur, often enhancing pharmacological activity without increasing toxicity [1, 39]. Selenium‐containing heterocycles have shown potential for treating cerebral ischemia, while selenocyanate derivatives have demonstrated vigorous antiparasitic activity against *Trypanosoma cruzi*with minimal toxicity [1, 40, 41]. These compounds represent promising therapeutic candidates for diseases such as diabetes, neurodegenerative disorders, and cancer, primarily through redox modulation via the thioredoxin system. Despite initial concerns regarding toxicity, many selenium‐containing molecules exhibit lower toxicity than the inorganic form of selenium, and their catalytic roles in oxidative reactions further highlight their medicinal and synthetic relevance.

A critical characteristic of organoselenium compounds is their high lipophilicity, which restricts their solubility primarily to organic solvents or oils. This property significantly limits their administration routes [11]. For example, Ebselen and its analogs, recognized for their antioxidant and anti‐inflammatory properties as glutathione peroxidase mimetics, have undergone several clinical trials [27, 28, 42]. In a clinical study evaluating Ebselen for acute ischemic stroke, the compound was administered orally as a bulk suspension in water, a nonoptimized dosage form [11, 42]. It has also been found that Ebselen has low selectivity in binding to reactive cysteines, as it is nucleophilically attached through the amino acid thiol functionality to the electrophilic selenium. However, several clinical trials have shown no signs of acute or long‐term toxicity [42].

Ebselen (2‐phenyl‐1,2‐benzisoselenazol‐3(2H)‐one) was first synthesized and characterized in the early 1980s as a synthetic organoselenium compound designed to mimic the activity of glutathione peroxidase (GPx). This key antioxidant enzyme detoxifies reactive oxygen species (ROS). Initially investigated for its antioxidant and anti‐inflammatory properties, Ebselen also demonstrated cytoprotective, neuroprotective, and antimicrobial activities, prompting early clinical interest in conditions such as ischemic stroke and noise‐induced hearing loss. Over the past 5 years, reviews have further highlighted its antioxidant properties (glutathione peroxidase‐mimetic activity), anti‐inflammatory effects (modulation of the NF‐κB and Nrf2/Keap1 pathways), antiviral action (covalent inhibition of the SARS‐CoV‐2 Mpro and PLpro proteases), neuroprotective effects (reduction of oxidative stress and preservation of synaptic integrity), and antidiabetic potential (improvement of insulin sensitivity via SHIP2 inhibition), establishing it as a multifunctional organoselenium compound of broad therapeutic relevance [43, 44, 45].

Similarly, diphenyl diselenide ((PhSe)_2_), another organoselenium compound, exhibits potent antioxidant and anti‐inflammatory activities and presents a solubility profile comparable to related selenium derivatives [28, 41, 46, 47, 48]. Luchese et al. (2013) reported that (PhSe)_2_reduces inflammation in a carrageenan‐induced pleurisy mouse model by inhibiting leukocyte infiltration, pleural exudation, and the production of pro‐inflammatory cytokines (TNF‐α, IL‐1β, IL‐6, and IFN‐γ), as well as by decreasing myeloperoxidase (MPO) activity. The antioxidant properties of (PhSe)_2_are closely associated, at least in part, with its anti‐inflammatory effects. However, (PhSe)_2_also displays strong plasma protein binding, requiring relatively high doses to reach pharmacologically active concentrations in biological tissues [49, 50]. Improving the aqueous solubility of such compounds could help overcome these pharmacokinetic limitations, expand possible administration routes, and enhance their therapeutic potential (Table 1).

**TABLE 1:** Summary of nanocarrier types reported in the last 10 years publications involving organoselenium compounds as cargo or carrier materials.

System	Used as	Formulation	Organoselenium compound	Ref
Nanocapsules	Drug delivery	Poly(ε‐caprolactone) Span 80 Medium chain triglycerides Tween 80 (polysorbate 80)	p,p’‐Methoxyl‐diphenyl diselenide 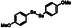	[51, 52]
Nanocapsules	Drug delivery	Canola oil, PCL, Span 80, MCT, Tween 80, PEG	Diphenyl diselenide 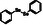	[53, 54, 55]
Nanospheres	Catalytic activity	Fmoc‐phenylalanine‐based selenide hydroperoxides (ROOH)	Phenylalanine‐based selenide 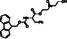	[56]
Nanoemulsion	Drug delivery	Cationic surfactants, *N*,*N*‐bis[3,3′‐(trimethylammonio)propyl] alkylamide dichlorides Oleic acid Mixed medium chain mono‐ and diglycerides propylene glycol monocaprylate (Capmul PG‐8)	2‐n‐Propylbenzisoselenazol‐3(2H)‐one (BSe) 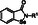	[57]
Nanoemulsion	Drug delivery	Captex 300 EP/NF, Kolliphor ELP tea tree oil	Ebselen 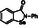	[58]
Micelles	Drug delivery	Tetronic 1107 (T1107) and Tetronic 904 (T904)	Selenodiazoles 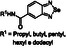	[59]
Micelles	Drug delivery	Organoselenium dendritic polymers	Selenium dendritic 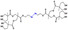	[60]
Micelles	Drug delivery	Amphiphilic block copolymer	Methylseleno‐aspirin 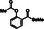	[61]
Nanoemulsion and micelles	Solubility	CTAB Tween 20 SDS	Fosforoselenides compounds 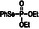	[62, 63]
Liposome	Drug delivery	HSPC/Egg PC/DOPE/mPEG2000‐ DSPE/cholesterol/DDA	Diselanediylbis decanoic acid (DDA) 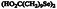	[64]

## 3 Major Interactions Between Organoselenium Compounds and Nanocarrier Systems

As stated in earlier sections, organoselenium compounds have attracted significant attention due to their broad spectrum of biological activities. However, their practical application is often limited by low solubility, instability, and unfavorable pharmacokinetics. To overcome these drawbacks, nanocarrier systems, such as liposomes [64], polymeric nanoparticles [65], micelles [59, 60], and dendrimers, have been explored as effective delivery platforms [11]. Nanocarriers are engineered structures that encapsulate, protect, and deliver bioactive molecules, thereby improving their stability, solubility, and targeted delivery. In this context, understanding the major interactions between organoselenium compounds and nanocarrier systems is essential, as these interactions dictate not only the efficiency of encapsulation and release but also the over all therapeutic performance (Table 1).

**FIGURE 3:**
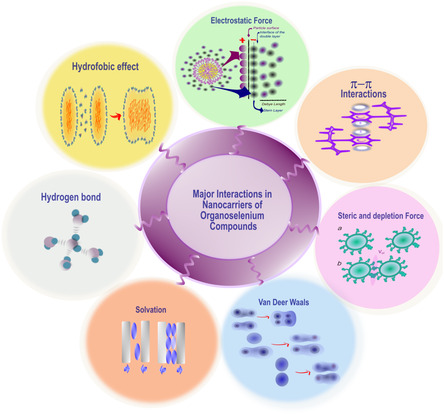
Description of the main forces involved in the stabilization of organoselenium compounds by nanocarriers. These forces depend on the nature of the stabilizing systems and the intrinsic properties of the organoselenium compounds.

The interactions governing the association between organoselenium compounds and nanocarrier systems are diverse and can significantly influence their physicochemical behavior and biological performance. Among them, the hydrophobic effect plays a central role, where the nonpolar domains of organoselenium molecules are stabilized within the carrier's hydrophobic core. In addition, hydrogen bonding and van der Waals interactions contribute to the stabilization of organoselenium compound‐nanocarrier systems, while electrostatic forces often dictate loading efficiency and release kinetics depending on the charge characteristics of both organoselenium and nanostructures (Figure 3). In some cases, stronger covalent bonds may be established, leading to more durable conjugation strategies. Surface adsorption phenomena can also occur, affecting organoselenium distribution at the nanocarrier interface. Together, these mechanisms provide a comprehensive framework for understanding how nanocarriers can be tailored to optimize the delivery of organoselenium compounds (Table 2). This section aims to discuss the fundamental types of interactions involved and how they enhance the biomedical applicability of organoselenium compounds.

**TABLE 2:** Physicochemical forces, assembly mechanisms, and functional performance across nanocarrier systems.

System	Dominant self‐assembly forces	Other significant contributions	Role in encapsulation/effect on performance	Ref.
**Micelles**	Hydrophobic driving force (solvent‐mediated) ∼ 3–10 kJ mol^−1^ per –CH_2_equivalent (size‐dependent); van der Waals (London dispersion ∼ 0.5–5 kJ mol^−1^(both scale with aggregate size)	Electrostatic (for ionic surfactants), hydrogen bonds (headgroups), solvation/ solvophobic effects, steric packing	Controls core formation and size, determines capacity for solubilizing lipophilic drugs; corona interactions determine colloidal stability	[63, 66, 67, 68]
**Emulsions/nanoemulsions**	Hydrophobic (oil–water immiscibility), van der Waals (Hamaker attraction between droplets; depend on Hamaker constant A and distance; Hamaker constants for organic systems ∼ 5–50 × 10^−20^J)	Steric/electrostatic repulsion from surfactant/polymer layers, interfacial tension, hydrogen bonding at interface	Determines droplet stability vs. coalescence, partitioning of active (oil vs water), interfacial loading influences release	[57, 58, 62]
**Polymeric nanocapsules/solid nanoparticles**	Hydrophobic interactions (for lipophilic actives), hydrogen bonds (polymer–drug, ∼5‐40 kJ mol^−1^typical ranges), van der Waals (dispersion cumulative can be tens of kJ mol^−1^per interacting motif)	π‐π stacking (aromatic organoselenium/polymers stacking energies vary ∼ 5–15 kJ·mol^−1^), electrostatic interactions (charged polymers), crosslinking (network rigidity)	Controls drug‐matrix affinity (entrapment efficiency), diffusional release, mechanical/structural retention	[53, 69, 70, 71]
**Adsorption (surfaces: silica, oxides, carbon)**	Van der Waals (universal), electrostatic (surface charge attraction/repulsion) can range from small to > 100 kJ mol^−1^depending on charge and separation, hydrogen bonding (surface OH)	Hydrophobic adsorption (on nonpolar surfaces), coordination/chemisorption (thiols, phosphates to metals), π‐π (graphitic surfaces)	Determines binding strength, selectivity, and capacity; strongly pH/ionic strength dependent	[72, 73]
**Liposomes (lipid vesicles)**	Hydrophobic effect (bilayer formation), van der Waals (lipid tail packing), hydration repulsion between bilayers	Electrostatic interactions (charged lipids), hydrogen bonding (headgroups), steric/PEG repulsion, bending elasticity (Helfrich energy)	Allows dual encapsulation: hydrophilic compounds in the aqueous core and hydrophobic molecules within the bilayer; bilayer rigidity/fluidity modulates loading, leakage, and release	[64, 74]

Hydrophobic interactions are critical for the encapsulation and stabilization of organoselenium compounds into amphiphilic carriers such as micelles, liposomes, and polymeric nanocapsules. This one refers to the aversion of nonpolar solutes to water, and organoselenium compounds are generally hydrophobic. These interactions minimize the system's free energy by sequestering organoselenium compounds within hydrophobic cores. When hydrophobic ligand‐coated NPs are dispersed in water, for example, hydrophobic interaction serves as an attractive force between NPs. A similar mechanism occurs in the formation of self‐assembly systems such as micelles, liposomes, and others. The hydrophobic environment within carriers reduces oxidative degradation of sensitive organoselenium compounds. In drug delivery, for example, encapsulation of organoselenium compounds such as Ebselen in polymeric micelles has demonstrated enhanced bioavailability and targeted release [59, 72], while lipid‐based carriers stabilize organoselenium compounds in physiological environments, extending their therapeutic half‐life.

The addition of an apolar molecule, like organoselenium compounds, in water, promotes the redistribution of ordered water molecules near apolar molecules and a consequent decrease of the Gibbs energy of the system, which is termed as hydrophobic interaction. This interaction occurs between hydrophobic groups such as benzene rings (π‐π stacking too) or hydrocarbon chains and the hydrophobic regions of biomolecules, with an energy range of 12–20 kJ mol^−1^. The mechanism of organoselenium compounds with nonpolar moieties involves interaction with the hydrophobic segments of surfactants or lipids, thereby promoting self‐assembly [62].

Beyond stabilizing organoselenium compounds, hydrophobic interactions also dictate the morphology and loading capacity of nanocarrier systems. Stronger hydrophobic interactions tend to produce more compact nanostructures with reduced surface energy, thereby improving colloidal stability and extending in vivo circulation time. Moreover, by modulating the hydrophobic–hydrophilic balance of carrier components, it is possible to fine‐tune the release kinetics of organoselenium compounds, allowing either sustained or stimuli‐responsive delivery. Importantly, the shielding provided by hydrophobic cores not only protects organoselenium compounds from hydrolysis and oxidation but also reduces premature leakage during systemic transport. Taken together, these effects highlight the central role of hydrophobic interactions in optimizing both the physicochemical properties of nanocarriers and the pharmacological performance of organoselenium compounds.

**FIGURE 4:**
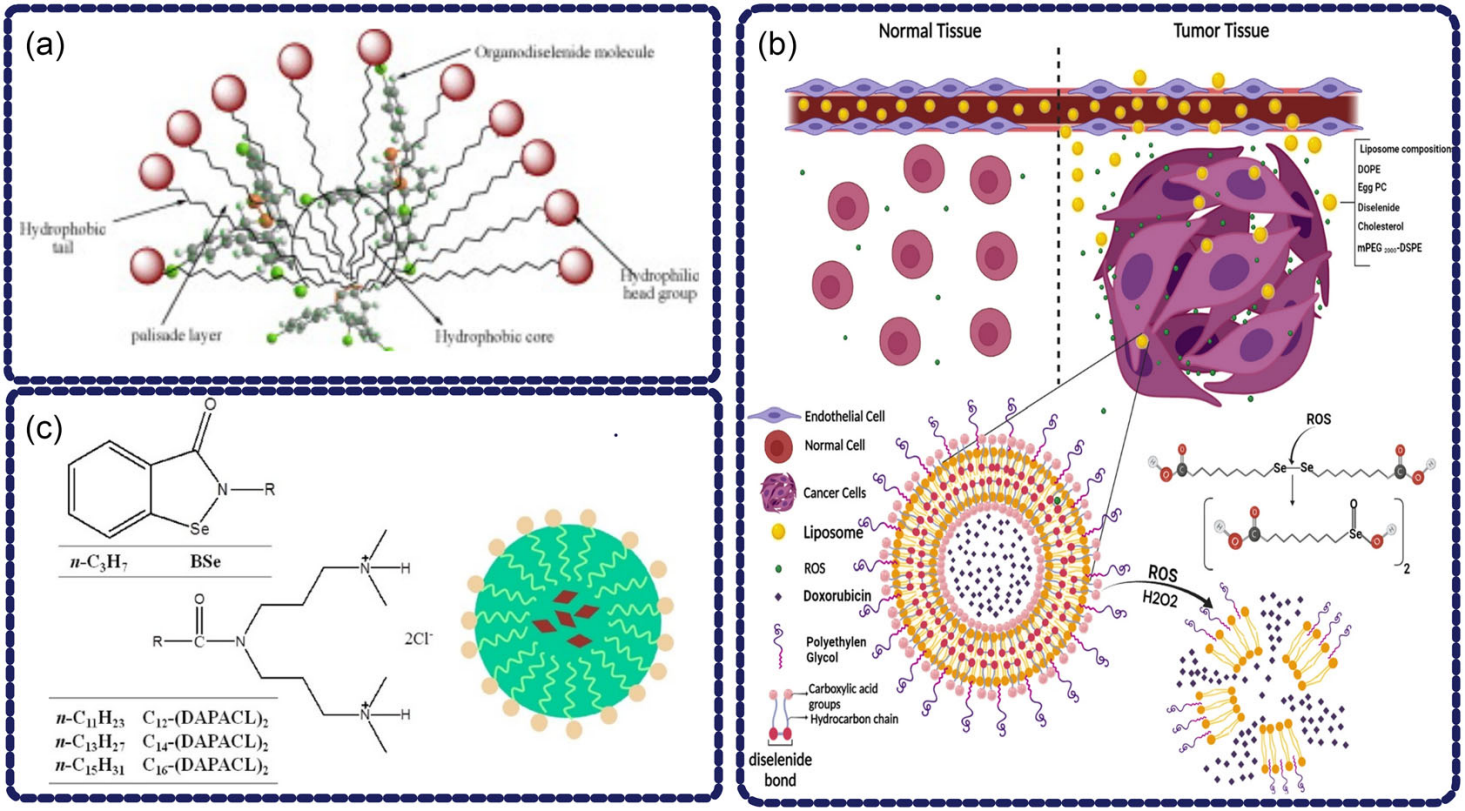
Representative physicochemical interactions between organoselenium compounds and nanocarrier systems. (a) Reprinted from [63]. Copyright (2011), with permission from Elsevier. (b) Reproduced from [74] licensed under CC BY 4.0. (c) Reprinted from [57]. Copyright (2016), with permission from Elsevier.

Figure 4 illustrates representative nanostructured systems in which hydrophobic interactions enable the incorporation of organoselenium compounds into amphiphilic carriers such as micelles, liposomes, and emulsions. In micellar systems, nonpolar diselenide derivatives preferentially localize in the hydrophobic core via interactions with surfactant alkyl chains, thereby stabilizing them in aqueous environments [63]. Comparable behavior occurs in liposomal formulations, where poorly water‐soluble organoselenium compounds partition into the lipid bilayer rather than the aqueous phase, occupying the hydrophobic domains of the membrane. Liposomes containing diselenide (Se—Se) bonds may further exhibit redox‐responsive behavior, since cleavage of the Se–Se linkage under specific redox conditions can trigger the release of the encapsulated drug [74]. Hydrophobic domains also play a key role in emulsion‐based systems, where organoselenium compounds are encapsulated in nanoemulsions prepared by rapid dilution and stabilized by bifunctional cationic surfactants. These formulations show high physical stability and improved bioavailability of the active compound, while enhancing antimicrobial activity against *Candida albicans* [57]. Collectively, these examples demonstrate how hydrophobic microenvironments within nanocarriers facilitate the incorporation and stabilization of organoselenium compounds, supporting their application in biomedical delivery systems.

Hydrogen bonding represents one of the most relevant noncovalent forces in the association between organoselenium compounds and nanocarriers, playing a pivotal role in stabilizing organoselenium compounds within polymeric matrices and surfactants. These interactions are significant because selenium‐containing groups, such as selenols, selenides, and selenoamides, can act as both hydrogen‐bond donors and acceptors depending on their chemical environment and oxidation state [75, 76]. The relatively high polarizability of selenium atoms enhances these interactions, facilitating the formation of directional hydrogen bonds with hydrophilic components of polymeric matrices [77].

In polymer‐based nanocarriers, hydrogen bonds often form between the drug's selenium moieties and polar groups such as hydroxyl, carboxyl, and amide within the polymer backbone. While selenium can participate in hydrogen bonding, it is not a primary mechanism for stabilizing nanocarriers through interactions with standard polar groups, which are far more likely to form hydrogen bonds with other electronegative parts of the encapsulated organoselenium compound. However, such interactions can increase encapsulation stability and reduce premature drug release by promoting intermolecular cohesion within the carrier matrix. The strength and persistence of hydrogen bonds can modulate the release profile of organoselenium compounds. Stronger or multiple hydrogen bonds tend to retard diffusion, leading to sustained release behavior, whereas weaker, transient interactions facilitate faster release under physiological conditions [78].

These noncovalent interactions are fundamental in maintaining molecular organization and controlling drug mobility, ultimately influencing the physicochemical and biological performance of organoselenium‐based systems. Through directional bonding, hydrogen bonds facilitate the self‐assembly of organoselenium compounds into organized supramolecular architectures, which can be harnessed for both therapeutic and catalytic applications. In micellar or liposomal systems, for example, hydrogen bonding between the organoselenium molecule and the polar headgroups of surfactants [62] or phospholipids may anchor the drug near the interface, influencing both its orientation and release dynamics [57].

Many organoselenium compounds possess ionizable groups or polarizable moieties, where their charge distribution can vary depending on the medium's pH, enabling dynamic interactions with charged polymers, surfactants, or inorganic surfaces. In such cases, electrostatic interactions govern the association of charged organoselenium compounds with oppositely charged carrier systems, providing an effective strategy for improving encapsulation, retention, and controlled release. For instance, SeNPs can interact strongly with positively charged polymers such as chitosan or quaternized chitosan derivatives, forming stable colloidal assemblies [79].

Importantly, the strength of electrostatic binding is highly sensitive to pH variations, which alter the protonation state of both the organoselenium compound and the carrier. Under acidic conditions, amino groups become protonated, strengthening interactions with negatively charged organoselenium compounds; at higher pH, deprotonation weakens these attractions and promotes molecular release. This property allows for pH‐triggered drug release, a key feature for site‐specific delivery in tumor environments [65]

Covalent attachment of organoselenium compounds to carrier matrices represents a robust and widely explored strategy to achieve long‐term stability, sustained release, and enhanced specificity. Unlike noncovalent interactions, covalent bonding provides a permanent linkage between organoselenium molecules and the carrier, minimizing premature leakage and improving pharmacokinetic control. This approach has been effectively used in therapeutic delivery systems, where covalent conjugation converts the carrier–organoselenium complex into a prodrug‐like platform, enabling bond cleavage under specific biological stimuli such as redox potential, pH variations, or enzymatic activity [80]. Recent studies illustrate this trend: selenide‐functionalized hydrogels have been engineered to provide ROS‐triggered degradation [81], and diselenide‐crosslinked polymeric micelles have shown enhanced stability, on‐demand cleavage, and improved delivery efficiency for hydrophobic drugs [66, 67]. Together, these systems highlight how covalent bonding enables precise control over organoselenium release kinetics and bioactivity, positioning covalently conjugated organoselenium‐carrier assemblies as a promising class of intelligent, stimuli‐responsive therapeutic platforms.

Charge transfer and chalcogen bonding interactions are increasingly recognized as key contributors to the stabilization and functionality of organoselenium compounds in encapsulated systems. While weaker than covalent bonds, these directional and tunable interactions confer selectivity and reversibility, enabling controlled self‐assembly, electronic modulation, and stimulus‐responsive release behaviors [82, 83]. Chalcogen bonds are noncovalent interactions involving electrophilic regions of chalcogen atoms (such as selenium) interacting with a Lewis base in the same molecule or with another molecular entity (e.g., O, N, or π‐systems). They are particularly relevant for organoselenium compounds, given selenium's capacity to act both as donor and acceptor in supramolecular architectures [84, 85]. Recent studies provide additional examples of functional applications: Sari et al. (2022) [11] demonstrated that organoselenium compounds formulated in nano‐based carriers enhance stability and controlled release, and Chauhan et al. [82] showed that chalcogen bonding can boost the cellular uptake of organoselenium molecules, highlighting its role in designing responsive nanocarrier systems.

Van der Waals interactions play a critical role in the stabilization and organizing organoselenium compounds within nanostructured carriers. These noncovalent forces, including London dispersion and dipole‐induced dipole interactions, become particularly significant in systems where covalent or electrostatic bonding is limited. Dispersion interactions arise from the transient polarization of electron clouds between adjacent molecules or molecular segments. In organoselenium‐loaded nanocarriers, these interactions primarily occur between the aromatic or aliphatic moieties of the organoselenium compounds and the hydrophobic domains or pore walls of the carrier material, thereby enhancing molecular packing, sustaining retention, and controlling release kinetics. Recent studies provide supporting evidence: Lorenzoni et al. [86] highlighted the role of hydrophobic and dispersive interactions in the stabilization of organoselenium‐functionalized nanoparticles; del Olmo et al. [60] demonstrated that the balance between hydrophobic and hydrophilic domains in dendritic polymers enhances retention and delivery efficiency of organoselenium compounds. These findings underscore the fundamental importance of Van der Waals and hydrophobic interactions in the design of nanocarrier systems for controlled organoselenium delivery.

Adsorption processes constitute an important strategy for incorporating organoselenium compounds into nanostructured and self‐assembled delivery systems. In porous hydrophobic surfaces—such as granular activated carbon—adsorption is predominantly governed by nonelectrostatic interactions, including van der Waals forces, hydrophobic interactions, and binding at localized surface sites. Experimental studies using selenocysteine and selenomethionine have demonstrated adsorption behaviors that fit both Langmuir and Freundlich isotherms and exhibit pseudo‐second‐order kinetics, confirming a mechanism driven by surface affinity rather than a chemical reaction [73]. In self‐assembled nanocarriers, hydrophobic domains, such as micellar cores, can act as reservoirs that stabilize organoselenium compounds through hydrophobic and dispersive interactions, thereby reducing their susceptibility to oxidative degradation and improving solubility. These micelles can subsequently be integrated into layer‐by‐layer (LbL) assemblies, where adsorption onto oppositely charged or chemically complementary layers occurs primarily via electrostatic attraction, hydrogen bonding, and hydrophobic contributions, depending on the nature of the polyelectrolytes employed. This combination of hydrophobic compartmentalization and multilayer structuring enables sustained release profiles and preserves the catalytic activity of organoselenium molecules, as reported for micelle‐embedded Ebselen coatings used in biomedical interfaces [72].

Taken together, the various interaction mechanisms governing the association of organoselenium compounds with nanocarrier systems define a finely tuned balance between stability, responsiveness, and functionality. Hydrogen bonding and van der Waals forces contribute to the initial stabilization and structural homogeneity of organoselenium compounds within polymeric and lipid matrices, supporting supramolecular organization and preventing aggregation. If electrostatic interactions occur, usually, enhanced encapsulation and pH‐sensitive release, particularly in polyelectrolyte and tumor‐targeted systems, are the driven mechanisms of stabilization. Charge transfer and chalcogen bonding interactions have emerged as sophisticated design tools that couple structural stabilization with electronic modulation, enabling the creation of redox‐responsive, catalytic, or photoactive materials.

Collectively, these diverse forces enable precise control over encapsulation efficiency, release kinetics, and bioactivity of organoselenium compounds, highlighting the central role of intermolecular chemistry in the design of advanced organoselenium‐based nanoplatforms. A comprehensive understanding of these interactions not only deepens the fundamental insight into organoselenium compound‐nanocarrier behavior but also provides a molecular framework for the rational design of multifunctional, adaptive, and biocompatible delivery systems aimed at therapeutic, diagnostic, and catalytic applications.

The intricate physicochemical interactions governing organoselenium compounds within nanocarrier systems not only determine the structural stability and encapsulation efficiency but also directly influence their functional performance. While understanding these forces provides insight into carrier design and stabilization, it is their downstream impact on biological outcomes that ultimately defines their utility. Consequently, evaluating how these interactions translate into bioavailability, pharmacokinetics, and potential toxicity is essential to assess the therapeutic potential and safety of organoselenium‐based nanostructures fully. The following section, therefore, focuses on the current knowledge of bioavailability and toxicological considerations, linking molecular‐level interactions to biological behavior.

## 4 Toxicity Versus Bioavailability

Selenium possesses a narrow therapeutic window, meaning that the difference between beneficial and toxic doses is small. Selenium deficiency is associated with oxidative stress and immune dysfunction, while excessive intake may lead to selenosis, a condition characterized by gastrointestinal disorders, alopecia, and neurological damage. This duality highlights the need for precise dose control and advanced formulation strategies in the development of organoselenium‐based therapies [19, 87].

In addition to toxicity concerns, poor bioavailability remains a major obstacle [19]. This limitation is further exacerbated by the intrinsic thiol reactivity of many synthetic organoselenium compounds, which readily form selenenyl‐sulfide intermediates with endogenous cysteine‐containing proteins. Such rapid thiol interactions not only contribute to off‐target toxicity but also diminish metabolic stability and reduce the fraction of intact compound available for absorption and systemic distribution, ultimately impairing adequate bioavailability [4]. Many organoselenium compounds exhibit low aqueous solubility, rapid metabolic degradation, and limited permeability across biological membranes, leading to suboptimal pharmacokinetics. Structural features such as molecular weight, lipophilicity, and susceptibility to enzymatic reduction strongly influence their absorption and systemic distribution [11, 88].

**FIGURE 5:**
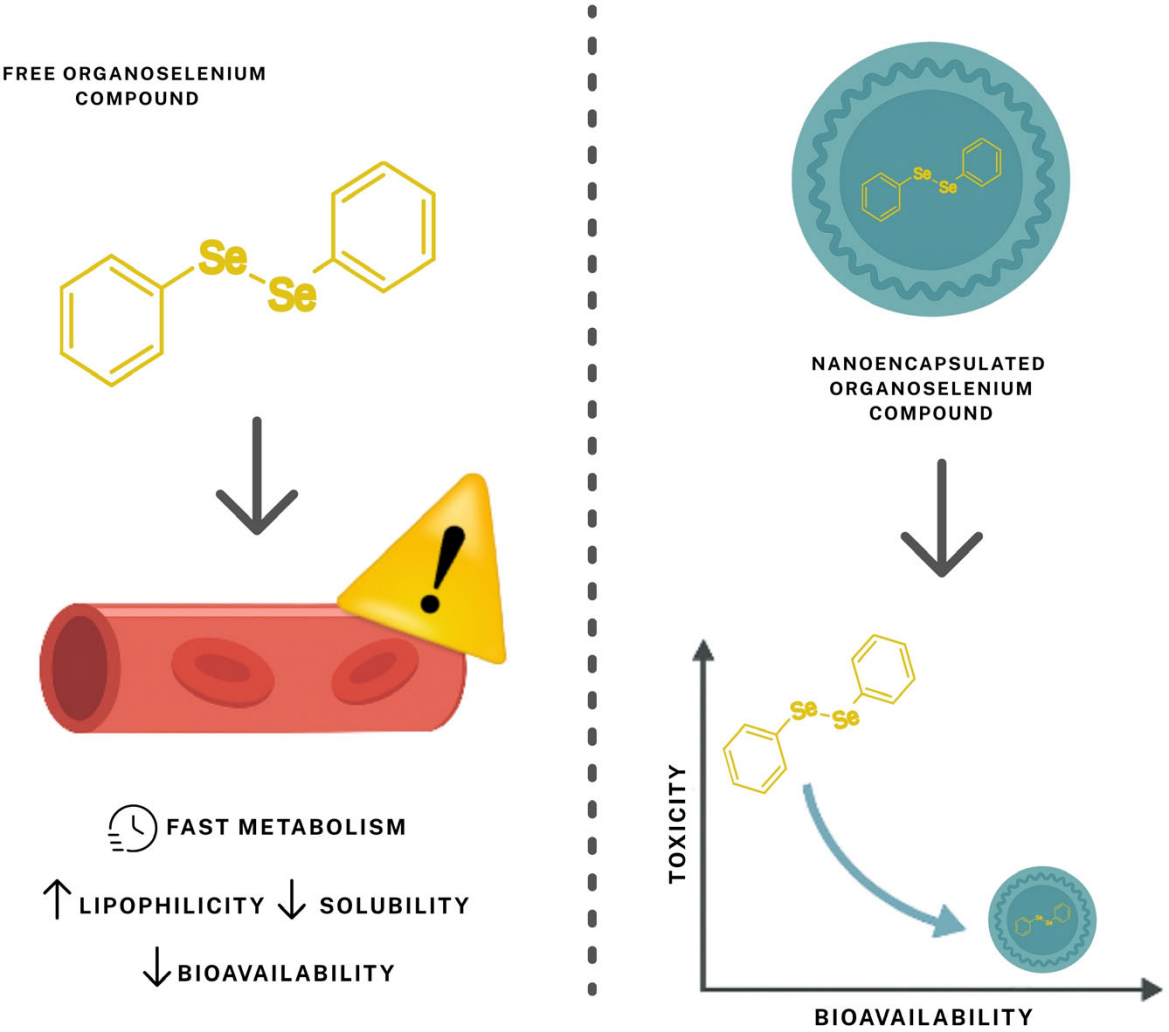
Scheme of the effect of nanoencapsulation on the toxicity and bioavailability of organoselenium compounds. In this figure, diphenyl diselenide was used as a model.

To address these limitations, nanocarrier‐based delivery systems have been widely explored for their ability to improve the pharmacokinetic profiles of organoselenium compounds, thereby increasing bioavailability while reducing systemic toxicity. As illustrated in the schematic example presented in Figure 5, nanoencapsulation can modulate key physicochemical and biological properties of these compounds, such as solubility, stability, metabolism rate, and toxicity.

For instance, polymeric nanocapsules containing the compound p,p′‐methoxyl‐diphenyl diselenide demonstrated chemical stability and did not induce toxicity in mice (no significant changes in body weight or organ histopathology). Moreover, these nanocapsules exhibited enhanced antioxidant activity compared with the free compound, suggesting greater therapeutic potential [52]. Another example involves Ebselen, which was incorporated into liposomes. The resulting formulation increased the compound's bioavailability and circulation time while significantly reducing systemic toxicity compared with free Ebselen, demonstrating the potential of nanocarriers to safely enhance the therapeutic efficacy of organoselenium compounds [89].

These findings highlight the central pharmacological challenge of organoselenium compounds: achieving an optimal balance between therapeutic efficacy and safety. From a physicochemical standpoint, parameters such as particle size, surface charge, and encapsulation efficiency critically influence biodistribution and toxicity [90, 91]. For instance, nanosystems within the 100–200 nm range tend to circulate longer and accumulate more efficiently in target tissues through enhanced permeability and retention, whereas larger or polydisperse particles may be rapidly cleared by the mononuclear phagocyte system [92, 93]. Surface charge also modulates biological interactions: positively charged nanocarriers generally exhibit greater cellular uptake. Still, they may increase nonspecific protein adsorption and cytotoxicity, while neutral or slightly negative surfaces often improve biocompatibility and systemic stability [94, 95]. Similarly, high encapsulation efficiency not only ensures sustained release of organoselenium compounds but also minimizes exposure to free selenium species, thereby reducing the risk of off‐target oxidative stress. Together, these physicochemical properties provide essential levers to fine‐tune redox reactivity, improve pharmacokinetics, and ultimately enhance the therapeutic window of organoselenium‐based nanomedicines [10, 96].

In support of these observations, several recent studies have demonstrated that nanostructured delivery systems can effectively improve the bioavailability of organoselenium compounds while simultaneously reducing their intrinsic toxicity. As summarized in Table 3, different nanocarriers have been shown to enhance key parameters, including solubility, metabolic stability, cellular uptake, and systemic distribution of these compounds. These nanostructured systems not only mitigate thiol‐related toxicity but also potentiate antioxidant, antimicrobial, antifungal, and anticancer activities, including in multidrug‐resistant cell models, reinforcing nanotechnology as an essential strategy to expand the therapeutic window of organoselenium compounds.

**TABLE 3:** Main findings on toxicology and bioavailability in models applied to nanocarriers of organoselenium compounds.

Compound	Nanocarrier type	Main results	Model	Ref.
p,p′‐Methoxyl‐diphenyl diselenide (OMe‐PhSe_2_)	Nanocapsules	Improved chemical stability, no toxicity in mice, enhanced antioxidant activity compared to free compound	In vivo	[51]
Ebselen	Polymer‐engineered liposomes	Enhanced solubility of Ebselen, prolonged circulation time, improved tumor accumulation and anticancer efficacy compared with free Ebselen; reduced systemic toxicity.	In vitro/in vivo	[97]
Ebselen	Silver nanoparticles	Improved antibacterial effect, enhanced anti‐inflammatory activity, inhibition of periodontal tissue destruction, and optimized therapeutic performance	In vitro/in vivo	[98]
Ebselen	Nanoemulsion	Increased antifungal activity; improved solubility and stability; enhanced penetration into fungal cells; reduced required therapeutic dose.	In vitro	[58]
Diphenyl diselenide	Nanocapsules	Selective antimelanoma activity in vitro; improved delivery to melanoma cells; reduced cytotoxicity to nontarget cells;	In vitro	[55]
Diselenide‐bridged	Mesoporous silica nanoparticles	Showed low systemic toxicity and high biocompatibility, with no significant adverse effects. Improved local bioavailability at the injury site while minimizing off‐target exposure.	In vivo	[99]
Organoselenium‐functionalized AuNPs	Gold nanoparticles	Enhanced stability and modulation of cytotoxicity. Covalent bonding minimized toxicity, while noncovalent functionalization increased anticancer activity.	In vitro	[86]
ACAT‐Se (organoselenium nucleoside analog)	pH‐sensitive PLGA nanoparticles	Enhanced antioxidant activity and cytotoxicity in drug‐resistant cancer cells while maintaining tumor selectivity and reducing off‐target toxicity. Improved cellular uptake and enabled co‐delivery with doxorubicin.	In vitro	[100]
ACAT‐Se (organoselenium nucleoside)	Transferrin‐decorated PLGA nanoparticles	Improved ACAT‐Se delivery with controlled release, higher cellular uptake, and greater cytotoxicity in 2D/3D tumor models. Also enhanced antioxidant activity, and showed hemocompatibility, resulting in higher efficacy and lower off‐target toxicity.	In vitro	[101]

## 5 Other Applications of Organoselenium Compounds

Organoselenium compounds have gained increasing relevance across several interdisciplinary domains beyond their well‐established antioxidant, anticancer, and anti‐inflammatory activities. Their versatile redox chemistry and structural tunability have enabled applications that span catalysis, materials science, virology, neuroprotection, agriculture, and surface engineering.

In green and sustainable chemistry, these compounds serve as valuable catalysts, promoting diverse oxidative and reductive transformations under mild, eco‐friendly conditions and broad compatibility with different functional groups. Both selenides and diselenides are efficient in asymmetric oxidations, epoxidations, and Baeyer‐Villiger reactions, often employing hydrogen peroxide or molecular oxygen as green oxidants [102]. Diphenyl diselenide, for instance, catalyzes the oxidation of alcohols and sulfides in aqueous media with remarkable selectivity [103], while organoselenium‐based catalysts often present recyclability and lower toxicity, reinforcing their potential as sustainable alternatives to heavy‐metal systems.

In addition to their direct catalytic activity, organoselenium motifs have been incorporated into catalytic systems as ligands or functional groups that coordinate transition metals. The soft donor character of selenium enables the formation of stable coordination environments with metals such as Pd, Cu, and Ru, thereby modulating catalytic reactivity and selectivity [104]. This strategy has been extended to heterogeneous catalysis, where selenium‐containing ligands are anchored to solid supports, such as silica or magnetic nanoparticles. In several reported systems, magnetic cores such as Fe_3_O_4_ are coated with silica and further functionalized with organic ligands containing selenium and amine donor groups, which coordinate catalytically active metals at the nanoparticle surface [105]. These core–shell architectures combine the catalytic activity of metal centers with the advantages of heterogeneous systems, including improved stability and facile catalyst recovery through magnetic separation [104, 105, 106, 107, 108].

In the biomedical field, several organoselenium derivatives have demonstrated antiviral activity through mechanisms that include interference with viral proteases and modulation of host oxidative stress. Ebselen is a notable example, capable of inhibiting the main protease of SARS‐CoV‐2 and thereby suppressing viral replication [109]. Compounds such as selenazoles and selenoureas have also shown broad‐spectrum antiviral activity against viruses like herpes simplex and influenza, primarily by disrupting redox‐sensitive viral enzymes [110]. These findings highlight the promise of organoselenium scaffolds for the development of multifunctional antiviral agents.

Organoselenium compounds’ relevance extend into neuroprotection, where specific organoselenium molecules interact with neurotransmission pathways, offering potential therapeutic value for neurodegenerative and neuropsychiatric conditions. Diphenyl diselenide and Ebselen modulate glutamatergic and GABAergic neurotransmission through redox‐dependent mechanisms, exerting neuroprotective and anticonvulsant effects in experimental models of epilepsy [4]. Additionally, newly designed selenium‐indole hybrids have shown inhibitory activity against monoamine oxidase B (MAO‐B), suggesting future utility in Parkinson's disease management [111].

In materials science and nanotechnology, selenium‐containing polymers and nanocomposites are being explored for applications in electronics, optics, and sensing. Their semiconducting behavior, combined with reversible redox transitions, enhances conductivity, photostability, and charge mobility in organic electronic devices [80]. Poly(selenophene)‐based conjugated polymers, for example, have been investigated in flexible solar cells and biosensors, often outperforming sulfur analogs. Selenium‐doped carbon nanomaterials are also under evaluation as electrocatalysts in energy storage and conversion systems [112].

In agriculture, organoselenium compounds are gaining attention as micronutrient supplements that enhance crop tolerance to abiotic stresses while improving nutritional value. Selenium‐enriched fertilizers have been shown to reduce oxidative damage and stimulate plant development under drought and salinity stress [113]. In animal nutrition, selenium‐enriched yeast and selenoamino acids are incorporated into feed to support antioxidant defenses, strengthen immune responses, and improve reproductive outcomes in livestock [114].

Finally, the integration of organoselenium moieties into polymeric coatings has yielded self‐sterilizing and antifouling materials suitable for biomedical and industrial applications. These selenium‐functionalized surfaces generate ROS in situ, preventing bacterial adhesion and suppressing biofilm formation [115]. Such coatings have been successfully applied to catheters, surgical tools, and water treatment membranes, providing durable antimicrobial protection without relying on leachable biocides [116]

These findings highlight the remarkable functional versatility of organoselenium compounds, whose redox plasticity and structural tunability enable applications ranging from catalysis and therapeutics to materials science and agricultural biotechnology. Although these advances represent significant progress, they also reveal unresolved challenges related to safety, mechanistic understanding, and translational feasibility. Addressing these gaps will be essential for guiding the next generation of organoselenium‐based technologies.

## 6 Summary and Outlook

This review examined the physicochemical forces governing the organization, stability, and performance of organoselenium‐nanocarrier systems, ranging from noncovalent interactions to covalent anchoring strategies. Hydrophobic interactions strongly influence the self‐assembly of both organoselenium cargos and amphiphilic building blocks, defining core–shell organization and promoting efficient encapsulation. Van der Waals forces modulate intra‐carrier packing and contribute to the cohesive integrity of the nanostructure. In addition, steric stabilization, electrostatic interactions, solvation forces, and hydration repulsion collectively maintain colloidal stability, preventing premature aggregation or phase separation. These interactions are also key determinants of the release profile, as they regulate molecular mobility and diffusion within the carrier matrix, enabling controlled or stimuli‐responsive delivery.

As a result, the integration of organoselenium compounds into nanocarrier systems has evolved from an emerging concept to an ongoing strategy with many advantages. Many studies have demonstrated that nanoformulations of organoselenium derivatives can enhance their physicochemical performance and improve solubility, stability, and biological availability while decreasing toxicity. However, beyond formulation improvements, a deeper understanding of the molecular interactions between organoselenium compounds and the nanocarrier matrix is a key factor. It seems a central topic to advancing this field, which is our central point of view in this review. These interactions govern encapsulation efficiency, release dynamics, and availability. Therefore, future work should not only report biological outcomes but also elucidate how intermolecular forces and nanoscale organization dictate system performance. Although pharmaceutical and therapeutic applications remain the main driving force for organoselenium‐based nanocarriers, these systems have also found relevance in broader domains such as catalysis and agricultural applications.

Integrating computational calculation and molecular modeling offers a compelling strategy to complement experimental formulation work [117]. Techniques such as molecular dynamics (MD) simulations can provide atomistic and meso‐scale insights into how a given organoselenium molecule might orient, partition, and interact within a candidate nanocarrier. Although we did not identify a study that explicitly applies this approach to organoselenium‐loaded nanocarriers, the literature provides successful precedents in hydrophobic drug systems, validating the concept and motivating its application in this context [118]. In the authors’ opinion, the focus could increasingly shift from empirical formulation toward a molecularly informed design of organoselenium‐encapsulated nanostructures, guided by both experimental characterization and computational insights. This perspective binds interfacial chemistry and nanoscale interaction.

As a promising eco‐friendly strategy, biosurfactants have emerged as amphiphilic agents for the design of environmentally sustainable nanocarriers capable of encapsulating lipophilic compounds [119, 120, 121]. These bio‐based molecules combine interfacial efficiency, biodegradability, and biocompatibility, providing new opportunities for the formulation of nanocarriers. Several recent studies have demonstrated this potential: mannose‐based surfactants efficiently stabilized oil‐in‐water nanoemulsions with low energy input, enhancing the dispersion of hydrophobic phases [122]; surfactant‐stabilized poly(D,L‐lactide) nanoparticles exhibited excellent colloidal stability and skin permeation with reduced cytotoxicity [123]; and triblock polymer–modified liposomes incorporating sodium cholate hydrate acting as a biosurfactant successfully co‐encapsulated hydrophilic and hydrophobic compounds, improving vesicle stability and permeability [124]. Together, these findings illustrate how biosurfactants can act as both structural and functional components in nanocarrier systems, as well as be biocompatible and sustainable, offering an alternative for new nanocarrier formulations. No reports currently describe the use of biosurfactants in combination with organoselenium compounds, highlighting a promising opportunity for further research.

Overall, the perspectives provided in this review highlight the relevance of physicochemical interactions as central drivers of functionality in organoselenium‐based nanocarriers. The size, surface chemistry, and morphology of the resulting nanocarriers substantially influence biological fate, including cellular uptake pathways, circulation time, and toxicity. Consequently, understanding the interplay of intermolecular forces in these systems is essential for rational design, optimization, and safe implementation. A deeper mechanistic understanding of these forces will be critical for guiding next‐generation strategies to enhance bioavailability, reduce toxicity, enable targeted delivery, and expand the scope of organoselenium applications across biomedical, catalytic, and multifunctional domains.

## Author Contributions

**Romelly Eugenia Rojas Ramírez**: conceptualization, formal analysis, investigation, writing – original draft, writing – review and editing. **Tielle Moraes de Almeida**: formal analysis, investigation, writing – original draft, writing – review and editing. **Daiani Canabarro Leite**: conceptualization, formal analysis, investigation, writing – original draft, writing – review and editing. **Gilson Zeni**: methodology, funding acquisition, supervision, writing – review and editing.

## Funding

This work was supported by Fundação de Amparo à Pesquisa do Estado do Rio Grande do Sul (grant 21/2551‐0002314‐7), Coordenação de Aperfeiçoamento de Pessoal de Nível Superior (grants PROEX# 88881.844988/2023‐01, AUXPE# 1333/2023), and Conselho Nacional de Desenvolvimento Científico e Tecnológico (grant 404471/2023‐4).

## Conflicts of Interest

The authors declare no conflicts of interest.

## Data Availability

Data sharing not applicable to this article as no datasets were generated or analyzed during the current study.

## References

[1] J. M. Sonego , S. I. De Diego , S. H. Szajnman , et al., “Organoselenium Compounds: Chemistry and Applications in Organic Synthesis,” Chemistry — A European Journal 29 (2023): e202300030. 37378970 10.1002/chem.202300030

[2] R. Do Carmo Pinheiro , L. Souza Marques , J. Ten Kathen Jung , et al., “Recent Progress in Synthetic and Biological Application of Diorganyl Diselenides,” The Chemical Record 24(2024): e202400044.38976862

[3] A. Madabeni , M. Bortoli , P. A. Nogara , et al., “50 Years of Organoselenium Chemistry, Biochemistry and Reactivity: Mechanistic Understanding, Successful and Controversial Stories,” Chemistry — A European Journal 30(2024): e202403003. 39304519 10.1002/chem.202403003 PMC11639659

[4] C. W. Nogueira , N. V. Barbosa , and J. B. T. Rocha , “Toxicology and Pharmacology of Synthetic Organoselenium Compounds: An Update,” Archives of Toxicology 95(2021): 1179–1226. 33792762 10.1007/s00204-021-03003-5 PMC8012418

[5] P. A. Nogara , M. E. Pereira , C. S. Oliveira , et al., “Organic Selenocompounds: Are They the Panacea for Human Illnesses?,” New Journal of Chemistry 47(2023): 9959–9988.

[6] A. Kunwar and K. I. Priyadarsini , History and Development of Selenium‐Based Radioprotectors: Distinctions between the Inorganic and Organic Forms,“ in Organoselenium Compounds in Biology and Medicine: Synthesis, Biological and Therapeutic Treatments, ed. V. K. Jain and K. I. Priyadarsini (Royal Society of Chemistry, 2017).

[7] C. Gallo‐Rodriguez and J. Rodriguez , “Organoselenium Compounds in Catalysis,” Synthesis 56, no. 12 (2024): 2295–2315, https://dpo.org/10.1055/a‐2197‐7356.

[8] D. Taylor , “The Pharmaceutical Industry and the Future of Drug Development,” in Pharmaceuticals in the Environment,(Royal Society of Chemistry, 2016), 1–33.

[9] G. Tiwari , R. Tiwari , B. Sriwastawa , et al., “Drug Delivery Systems: An Updated Review,” International Journal of Pharmaceutical Investigation 2(2012): 2–11. 23071954 10.4103/2230-973X.96920 PMC3465154

[10] H. Lu , J. Wang , T. Wang , et al., “Recent Progress on Nanostructures for Drug Delivery Applications,” Journal of Nanomaterials 2016(2016): 5762431.

[11] M. H. M. Sari , L. M. Ferreira , V. C. Prado , et al., “Nano‐Based Formulations as an Approach for Providing a Novel Identity for Organoselenium Compounds,” European Journal of Pharmaceutics and Biopharmaceutics 178(2022): 69–81. 35932964 10.1016/j.ejpb.2022.07.018

[12] A. Malhotra and J. N. Coupland , “The Effect of Surfactants on the Solubility, Zeta Potential, and Viscosity of Soy Protein Isolates,” Food Hydrocolloids 18(2004): 101–108.

[13] K. Pawar , R. Jayaram , and S. Bhagwat , “The Solubilization of Diphenyl Diselenide in Surfactant Solutions,” Journal of Dispersion Science and Technology 44(2021): 1–7.

[14] S. K. Mehta , S. Chaudhary , R. Kumar , et al., “Facile Solubilization of Organochalcogen Compounds in Mixed Micelle Formation of Binary and Ternary Cationic−Nonionic Surfactant Mixtures,” Journal of Physical Chemistry B 113(2009): 7188–7193. 19397306 10.1021/jp811310f

[15] K. K. Karthik , B. V. Cheriyan , S. Rajeshkumar , et al., “A Review on Selenium Nanoparticles and Their Biomedical Applications,” Biomedical Technology 6(2024): 61–74.

[16] L. Osorno , A. Brandley , D. Maldonado , et al., “Review of Contemporary Self‐Assembled Systems for the Controlled Delivery of Therapeutics in Medicine,” Nanomaterials 11(2021): 278. 33494400 10.3390/nano11020278 PMC7911285

[17] M. P. Rayman , “Selenium and Human Health,” The Lancet 379(2012): 1256–1268. 10.1016/S0140-6736(11)61452-9 22381456

[18] K. H. Winther , M. P. Rayman , S. J. Bonnema , et al., “Selenium in Thyroid Disorders—essential Knowledge for Clinicians,” Nature Reviews Endocrinology 16(2020): 165–176. 10.1038/s41574-019-0311-6 32001830

[19] G. Barchielli , A. Capperucci , and D. Tanini , “The Role of Selenium in Pathologies: An Updated Review,” Antioxidants 11(2022): 251. 35204134 10.3390/antiox11020251 PMC8868242

[20] D. Liang , C. Liu , and X. Zhang , “Association between Dietary Selenium Intake and the Risk of Cardiovascular Disease in US Adults: A Population‐Based Study,” Scientific Reports 15(2025): 13427. 40251378 10.1038/s41598-025-97867-7 PMC12008176

[21] A. Dominiak , A. Wilkaniec , P. Wroczyński , et al., “Selenium in the Therapy of Neurological Diseases. Where Is It Going?,” Current Neuropharmacology 14(2016): 282–299. 26549649 10.2174/1570159X14666151223100011 PMC4857624

[22] D. L. Hatfield , P. A. Tsuji , B. A. Carlson , et al., “Selenium and Selenocysteine: Roles in Cancer, Health, and Development,” Trends in Biochemical Sciences 39(2014): 112–120. 24485058 10.1016/j.tibs.2013.12.007 PMC3943681

[23] N. T. Le , Y. T.‐H. Pham , C. T.‐K. Le , et al., “A U‐Shaped Association between Selenium Intake and Cancer Risk,” Scientific Reports 14(2024): 21378. 39271688 10.1038/s41598-024-66553-5 PMC11399399

[24] M. Harthill , “Review: Micronutrient Selenium Deficiency Influences Evolution of Some Viral Infectious Diseases,” Biological Trace Element Research 143(2011): 1325–1336. 21318622 10.1007/s12011-011-8977-1 PMC7090490

[25] P. A. Tran and T. J. Webster , “Selenium Nanoparticles Inhibit Staphylococcus Aureus Growth,” International Journal of Nanomedicine 6(2011): 1553–1558. 21845045 10.2147/IJN.S21729 PMC3152473

[26] V. A. Potapov , M. V. Musalov , M. V. Musalova , et al., “Recent Advances in Organochalcogen Synthesis Based on Reactions of Chalcogen Halides with Alkynes and Alkenes,” Current Organic Chemistry 20(2015): 136–145.

[27] N. V. Barbosa , C. W. Nogueira , P. A. Nogara , et al., “Organoselenium Compounds as Mimics of Selenoproteins and Thiol Modifier Agents,” Metallomics 9(2017): 1703–1734. 29168872 10.1039/c7mt00083a

[28] M. Prigol , C. W. Nogueira , G. Zeni , et al., “In Vitro Metabolism of Diphenyl Diselenide in Rat Liver Fractions. Conjugation with GSH and Binding to Thiol Groups,” Chemico‐Biological Interactions 200(2012): 65–72. 23022272 10.1016/j.cbi.2012.09.007

[29] C. W. Nogueira , F. C. Meotti , E. Curte , et al., “Investigations into the Potential Neurotoxicity Induced by Diselenides in Mice and Rats,” Toxicology 183(2003): 29–37. 12504340 10.1016/s0300-483x(02)00423-7

[30] H. Sies and M. J. Parnham , “Potential Therapeutic use of Ebselen for COVID‐19 and Other Respiratory Viral Infections,” Free Radical Biology and Medicine 156(2020): 107–112. 32598985 10.1016/j.freeradbiomed.2020.06.032 PMC7319625

[31] Z. Chen , H. Lai , L. Hou , and T. Chen , “Rational Design and Action Mechanisms of Chemically Innovative Organoselenium in Cancer Therapy,” Chemical Communications 56(2020): 179–196. 10.1039/c9cc07683b 31782422

[32] R. Wang , H. Da , H. Wang , S. Ji , and Z. Tian , “Selenium Functionalized Carbon for High Dispersion of Platinum–ruthenium Nanoparticles and Its Effect on the Electrocatalytic Oxidation of Methanol,” Journal of Power Sources 233(2013): 326–330.

[33] B. Fan , F. Lin , X. Wu , Z. Zhu , and A. K.‐Y. Jen , “Selenium‐Containing Organic Photovoltaic Materials,” Accounts of Chemical Research 54(2021): 3906–3916. 34606230 10.1021/acs.accounts.1c00443

[34] M. Sak , Y. S. Al‐Faiyz , H. Elsawy , and S. Shaaban , “Novel Organoselenium Redox Modulators with Potential Anticancer, Antimicrobial, and Antioxidant Activities,” Antioxidants 11(2022): 1231. 35883724 10.3390/antiox11071231 PMC9312238

[35] K. Obata , T. Higashi , M. Hasegawa , M. Katayama , and K. Takanabe , “Synthesis of Metal Chalcogenide Semiconductors by Thermal Decomposition of Organosulfur and Organoselenium Compounds,” Chemistry — A European Journal 28(2022): e202201951. 35931660 10.1002/chem.202201951 PMC9804685

[36] R. B. da Silva , F. L. Coelho , H. de Castro Silva Junior , et al., “Organosulfur and Organoselenium Functionalized Benzimidazo[1,2‐a]quinolines: From Experimental and Theoretical Photophysics to All‐Solution‐Processed OLEDs,” Journal of Fluorescence 34(2024): 1427–1439. 37542587 10.1007/s10895-023-03358-1

[37] S. Panda , A. Panda , and S. S. Zade , “Organoselenium Compounds as Fluorescent Probes,” Coordination Chemistry Reviews 300(2015): 86–100.

[38] S. Lai , X. Liang , and Q. Zeng , “Recent Progress in Synthesis and Application of Chiral Organoselenium Compounds,” Chemistry — A European Journal 30(2024): e202304067. 38078625 10.1002/chem.202304067

[39] B. Kaya , M. G. Azad , M. Suleymanoglu , et al., “Isosteric Replacement of Sulfur to Selenium in a Thiosemicarbazone: Promotion of Zn(II) Complex Dissociation and Transmetalation to Augment Anticancer Efficacy,” Journal of Medicinal Chemistry 67(2024): 12155–12183. 38967641 10.1021/acs.jmedchem.4c00884

[40] P. Pyka , W. Haberek , M. Więcek , et al., “First‐in‐Class Selenium‐Containing Potent Serotonin Receptor 5‐HT6 Agents with a Beneficial Neuroprotective Profile against Alzheimer's Disease,” Journal of Medicinal Chemistry 67(2024): 1580–1610. 38190615 10.1021/acs.jmedchem.3c02148 PMC10823479

[41] R. Martín‐Escolano , D. Molina‐Carreño , D. Plano , et al., “Library of Selenocyanate and Diselenide Derivatives as In Vivo Antichagasic Compounds Targeting Trypanosoma Cruzi Mitochondrion,” Pharmaceuticals 14(2021): 419. 34062791 10.3390/ph14050419 PMC8147293

[42] C. Santi , C. Scimmi , and L. Sancineto , “Ebselen and Analogues: Pharmacological Properties and Synthetic Strategies for Their Preparation,” Molecules 26(2021): 4230. 34299505 10.3390/molecules26144230 PMC8306772

[43] Z. Polianskyte‐Prause , T. A. Tolvanen , S. Lindfors , et al., “Ebselen Enhances Insulin Sensitivity and Decreases Oxidative Stress by Inhibiting SHIP2 and Protects from Inflammation in Diabetic Mice,” International Journal of Biological Sciences 18(2022): 1852–1864. 35342343 10.7150/ijbs.66314 PMC8935241

[44] S. N. De Luca , K. Brassington , S. M. H. Chan , et al., “Ebselen Prevents Cigarette Smoke‐Induced Cognitive Dysfunction in Mice by Preserving Hippocampal Synaptophysin Expression,” Journal of Neuroinflammation 19(2022): 72. 35351173 10.1186/s12974-022-02432-y PMC8966248

[45] M. Zmudzinski , W. Rut , K. Olech , et al., “Ebselen Derivatives Inhibit SARS‐CoV‐2 Replication by Inhibition of Its Essential Proteins: PLpro and Mpro Proteases, and nsp14 Guanine N7‐Methyltransferase,” Scientific Reports 13(2023): 9161. 37280236 10.1038/s41598-023-35907-w PMC10242237

[46] M. Prigol , C. W. Nogueira , G. Zeni , M. R. Bronze , and L. Constantino , “Physicochemical and Biochemical Profiling of Diphenyl Diselenide,” Applied Biochemistry and Biotechnology 169(2013): 885–893. 23292905 10.1007/s12010-012-0042-9

[47] C. Luchese , M. Prigol , M. Duarte , and C. Nogueira , “Diphenyl Diselenide Reduces Inflammation in the Mouse Model of Pleurisy Induced by Carrageenan: Reduction of pro‐Inflammatory Markers and Reactive Species Levels,” Inflammation Research 61(2012): 1117–1124. 22699806 10.1007/s00011-012-0504-0

[48] R. M. Rosa , R. Roesler , A. L. Braga , J. Saffi , and J. A. P. Henriques , “Pharmacology and Toxicology of Diphenyl Diselenide in Several Biological Models,” Brazilian Journal of Medical and Biological Research 40(2007): 1287–1304. 18572457 10.1590/s0100-879x2006005000171

[49] J. Saluk , M. Bijak , P. Nowak , and B. Wachowicz , “Evaluating the Antioxidative Activity of Diselenide Containing Compounds in Human Blood,” Bioorganic Chemistry 50(2013): 26–33. 23941993 10.1016/j.bioorg.2013.07.003

[50] C. W. Nogueira and J. B. T. Rocha , “Diphenyl Diselenide a Janus‐Faced Molecule,” Journal of the Brazilian Chemical Society 21(2010): 2055–2071.

[51] M. H. M. Sari , L. M. Ferreira , V. Angonesi Zborowski , et al., “p,p’‐Methoxyl‐Diphenyl Diselenide Incorporation into Polymeric Nanocapsules Improves Its Antinociceptive Action: Physicochemical and Behavioral Studies,” Colloids and Surfaces B: Biointerfaces 157(2017): 464–472. 28651193 10.1016/j.colsurfb.2017.06.016

[52] M. H. Marcondes Sari , V. A. Zborowski , L. M. Ferreira , et al., “p,p′‐Methoxyl‐Diphenyl Diselenide‐Loaded Polymeric Nanocapsules as a Novel Approach to Inflammatory Pain Treatment: Behavioral, Biochemistry and Molecular Evidence,” European Journal of Pharmaceutical Sciences 111(2018): 38–45. 28943444 10.1016/j.ejps.2017.09.030

[53] L. M. Ferreira , M. H. M. Sari , V. F. Cervi , et al., “Design of Pegylated‐Nanocapsules to Diphenyl Diselenide Administration: In Vitro Evidence of Hemocompatible and Selective Antiglioma Formulation,” AAPS PharmSciTech 21(2020): 307. 33151442 10.1208/s12249-020-01845-3

[54] E. S. Zimmermann , L. M. Ferreira , L. B. Denardi , et al., “Mucoadhesive Gellan Gum Hydrogel Containing Diphenyl Diselenide‐Loaded Nanocapsules Presents Improved Anti‐Candida Action in a Mouse Model of Vulvovaginal Candidiasis,” European Journal of Pharmaceutical Sciences 167(2021): 106011. 34537375 10.1016/j.ejps.2021.106011

[55] L. M. Ferreira , V. F. Cervi , M. H. M. Sari , et al., “Diphenyl Diselenide Loaded Poly(ε‐Caprolactone) Nanocapsules with Selective Antimelanoma Activity: Development and Cytotoxic Evaluation,” Materials Science and Engineering C 91(2018): 1–9. 30033235 10.1016/j.msec.2018.05.014

[56] Z. Huang , Q. Luo , S. Guan , et al., “Redox Control of GPx Catalytic Activity through Mediating Self‐Assembly of Fmoc‐Phenylalanine Selenide into Switchable Supramolecular Architectures,” Soft Matter 10(2014): 9695–9701. 25366375 10.1039/c4sm02030h

[57] M. Piętka‐Ottlik , A. Lewińska , A. Jaromin , et al., “Antifungal Organoselenium Compound Loaded Nanoemulsions Stabilized by Bifunctional Cationic Surfactants,” Colloids and Surfaces A: Physicochemical and Engineering Aspects 510(2016): 53–62.

[58] S. Menon , R. Vartak , K. Patel , and B. Billack , “Evaluation of the Antifungal Activity of an Ebselen‐Loaded Nanoemulsion in a Mouse Model of Vulvovaginal Candidiasis,” Nanomedicine: Nanotechnology, Biology and Medicine 37(2021): 102428. 34217850 10.1016/j.nano.2021.102428

[59] A. Úriz , C. Sanmartín , D. Plano , et al., “Activity Enhancement of Selective Antitumoral Selenodiazoles Formulated with Poloxamine Micelles,” Colloids and Surfaces B: Biointerfaces 170(2018): 463–469. 29960214 10.1016/j.colsurfb.2018.06.009

[60] N. Sanz Del Olmo , J. San Jacinto García , Y. Yin , et al., “Responsive Organoselenium Dendritic Polymers: From Monodisperse Dendrimers to Self‐Assembled Micelles for Advanced Therapeutic Applications,” Journal of the American Chemical Society 147(2025): 18626–18636. 40407783 10.1021/jacs.5c00811 PMC12147156

[61] A. C. Ruberte , G. González‐Gaitano , A. K. Sharma , et al., “New Formulation of a Methylseleno‐Aspirin Analog with Anticancer Activity Towards Colon Cancer,” International Journal of Molecular Sciences 21(2020): 9017. 33260948 10.3390/ijms21239017 PMC7730823

[62] R. E. Rojas Ramírez , D. Canabarro Leite , A. M. S. Recchi , et al., “Novel Spontaneous Nanoemulsions for Phosphorus Selenide Compounds: Toward Enhanced Solubility and Stable Aqueous Formulations,” ACS Omega 10(2025): 56566–56577. 41322646 10.1021/acsomega.5c08867 PMC12658836

[63] S. K. Mehta and S. Chaudhary , “Micropartitioning and Solubilization Enhancement of 1,2‐Bis(bis(4‐Chlorophenyl) Methyl)diselane in Mixed Micelles of Binary and Ternary Cationic‐Nonionic Surfactant Mixtures,” Colloids and Surfaces B: Biointerfaces 83(2011): 139–147. 21145714 10.1016/j.colsurfb.2010.11.011

[64] S. Khabbazian , E. Mirhadi , F. Gheybi , et al., “Liposomal Delivery of Organoselenium‐Cisplatin Complex as a Novel Therapeutic Approach for Colon Cancer Therapy,” Colloids and Surfaces B: Biointerfaces 242(2024): 114085. 39018910 10.1016/j.colsurfb.2024.114085

[65] D. Mathes , L. B. Macedo , T. B. Pieta , et al., “Co‐Delivery of an Innovative Organoselenium Compound and Paclitaxel by pH‐Responsive PCL Nanoparticles to Synergistically Overcome Multidrug Resistance in Cancer,” Pharmaceutics 16(2024): 590. 38794252 10.3390/pharmaceutics16050590 PMC11124783

[66] V. G. Deepagan , S. Kwon , D. G. You , et al., “In Situ Diselenide‐Crosslinked Polymeric Micelles for ROS‐Mediated Anticancer Drug Delivery,” Biomaterials 103(2016): 56–66. 27372421 10.1016/j.biomaterials.2016.06.044

[67] X. Cheng , H. Li , X. Sun , et al., “Visible‐Light‐Induced Diselenide‐Crosslinked Polymeric Micelles for ROS‐Triggered Drug Delivery,” Molecules 29(2024): 3970. 39203048 10.3390/molecules29163970 PMC11357037

[68] C. Wei , Y. Zhang , H. Xu , et al., “Well‐Defined Labile Diselenide‐Centered Poly(ε‐Caprolactone)‐Based Micelles for Activated Intracellular Drug Release,” Journal of Materials Chemistry B 4(2016): 5059–5067. 32264032 10.1039/c6tb01040g

[69] F. Soares , S. T. Stefanello , F. Dobrachinski , et al., “Free Radical Scavenging In Vitro and Biological Activity of Diphenyl Diselenide‐Loaded Nanocapsules: DPDS‐NCS Antioxidant and Toxicological Effects,” International Journal of Nanomedicine 10(2015): 5663–5674. 26379436 10.2147/IJN.S87190 PMC4567224

[70] M. H. Marcondes Sari , L. M. Ferreira , V. A. Zborowski , et al., “p,p′‐Methoxyl‐Diphenyl Diselenide‐Loaded Polymeric Nanocapsules Are Chemically Stable and Do Not Induce Toxicity in Mice,” European Journal of Pharmaceutics and Biopharmaceutics 117(2017): 39–48. 28363598 10.1016/j.ejpb.2017.03.018

[71] O. Dos Reis Antunes Junior , E. Antônio , R. M. Mainardes , et al., “Preparation, Physicochemical Characterization and Antioxidant Activity of Diphenyl Diselenide‐Loaded Poly(lactic Acid) Nanoparticles,” Journal of Trace Elements in Medicine and Biology 39(2017): 176–185. 27908412 10.1016/j.jtemb.2016.09.010

[72] L. Yang , L.‐H. Li , L. Jiang , et al., “Micelle‐Embedded Coating with Ebselen for Nitric Oxide Generation,” Medical Gas Research 9(2019): 176–183. 31898602 10.4103/2045-9912.273955 PMC7802419

[73] S. O. Okonji , L. Yu , J. A. Dominic , et al., “Adsorption by Granular Activated Carbon and Nano Zerovalent Iron from Wastewater: A Study on Removal of Selenomethionine and Selenocysteine,” Water 13(2021): 23.

[74] E. Mirhadi , M. Mashreghi , A. Askarizadeh , et al., “Redox‐Sensitive Doxorubicin Liposome: A Formulation Approach for Targeted Tumor Therapy,” Scientific Reports 12(2022): 11310. 35788647 10.1038/s41598-022-15239-x PMC9253031

[75] K. K. Mishra , S. K. Singh , P. Ghosh , et al., “The Nature of Selenium Hydrogen Bonding: Gas Phase Spectroscopy and Quantum Chemistry Calculations,” Physical Chemistry Chemical Physics 19(2017): 24179–24187. 28840208 10.1039/c7cp05265k

[76] T. I. Madzhidov and G. A. Chmutova , “The Nature of Hydrogen Bonds with Divalent Selenium Compounds,” Journal of Molecular Structure: THEOCHEM 959(2010): 1–7.

[77] A. Chand , D. K. Sahoo , A. Rana , et al., “The Prodigious Hydrogen Bonds with Sulfur and Selenium in Molecular Assemblies, Structural Biology, and Functional Materials,” Accounts of Chemical Research 53(2020): 1580–1592. 32677432 10.1021/acs.accounts.0c00289

[78] D. Singh , “A Pioneer Review on Intermolecular Hydrogen Bonding in Drug‐Polymer Mixtures: Implication of a Stable Delivery System,” Journal of Macromolecular Science Part B 64(2024): 1–17.

[79] A. Lunkov , M. Konovalova , B. Shagdarova , et al., “Synthesis of Selenium Nanoparticles Modified by Quaternary Chitosan Covalently Bonded with Gallic Acid,” Polymers 15(2023): 2123. 37177269 10.3390/polym15092123 PMC10180991

[80] Q. Li , Y. Zhang , Z. Chen , et al., “Organoselenium Chemistry‐Based Polymer Synthesis,” Organic Chemistry Frontiers 7(2020): 2815–2841.

[81] L. Wang , K. Zhu , W. Cao , et al., “ROS‐Triggered Degradation of Selenide‐Containing Polymers Based on Selenoxide Elimination,” Polymer Chemistry 10(2019): 2039–2046.

[82] E. Chauhan , D. Giri , V. Govindaraj , and G. Mugesh , “Chalcogen Bonding Boosts the Uptake of Small Molecules in Mammalian Cells,” Angewandte Chemie International Edition 64(2025): e202511786. 40525643 10.1002/anie.202511786

[83] S. P. Thomas , K. Satheeshkumar , G. Mugesh , and T. N. Guru Row , “Unusually Short Chalcogen Bonds Involving Organoselenium: Insights into the Se–N Bond Cleavage Mechanism of the Antioxidant Ebselen and Analogues,” Chemistry – A European Journal 21(2015): 6793–6800. 25766307 10.1002/chem.201405998

[84] T. Fellowes , M. A. Sani , and J. M. White , “Fingerprints of Chalcogen Bonding Revealed Through 77Se NMR,” Chemistry – A European Journal 30(2024): e202400385. 38506412 10.1002/chem.202400385

[85] L. Vogel , P. Wonner , and S. M. Huber , “Chalcogen Bonding: An Overview,” Angewandte Chemie International Edition 58(2019): 1880–1891. 30225899 10.1002/anie.201809432

[86] S. Lorenzoni , S. Cerra , E. Angulo‐Elizari , et al., “Organoselenium Compounds as Functionalizing Agents for Gold Nanoparticles in Cancer Therapy,” Colloids and Surfaces B: Biointerfaces 219(2022): 112828. 36108370 10.1016/j.colsurfb.2022.112828

[87] S. L. DeAngelo , B. Győrffy , M. Koutmos , et al., “Selenoproteins and tRNA‐Sec: Regulators of Cancer Redox Homeostasis,” Trends in Cancer 9(2023): 1006–1018. 37716885 10.1016/j.trecan.2023.08.003 PMC10843386

[88] K. N. Sands , T. A. Tuck , and T. G. Back , “Cyclic Seleninate Esters, Spirodioxyselenuranes and Related Compounds: New Classes of Biological Antioxidants that Emulate Glutathione Peroxidase,” Chemistry – A European Journal 24(2018): 9714–9728. 29542192 10.1002/chem.201800182

[89] R. Fang , R. H. Bogner , S. L. Nail , et al., “Stability of Freeze‐Dried Protein Formulations: Contributions of Ice Nucleation Temperature and Residence Time in the Freeze‐Concentrate,” Journal of Pharmaceutical Sciences 109(2020): 1896–1904. 32112825 10.1016/j.xphs.2020.02.014

[90] A. Albanese , P. S. Tang , and W. C. W. Chan , “The Effect of Nanoparticle Size, Shape, and Surface Chemistry on Biological Systems,” Annual Review of Biomedical Engineering 14(2012): 1–16. 10.1146/annurev-bioeng-071811-150124 22524388

[91] A. E. Nel , L. Mädler , D. Velegol , et al., “Understanding Biophysicochemical Interactions at the Nano–bio Interface,” Nature Materials 8(2009): 543–557. 19525947 10.1038/nmat2442

[92] H. Maeda , “Toward a Full Understanding of the EPR Effect in Primary and Metastatic Tumors as Well as Issues Related to Its Heterogeneity,” Advanced Drug Delivery Reviews 91(2015): 3–6. 25579058 10.1016/j.addr.2015.01.002

[93] E. Blanco , H. Shen , and M. Ferrari , “Principles of Nanoparticle Design for Overcoming Biological Barriers to Drug Delivery,” Nature Biotechnology 33(2015): 941–951. 10.1038/nbt.3330 PMC4978509 26348965

[94] E. Fröhlich , “The Role of Surface Charge in Cellular Uptake and Cytotoxicity of Medical Nanoparticles,” International Journal of Nanomedicine 7(2012): 5577–5591. 23144561 10.2147/IJN.S36111 PMC3493258

[95] S. Wilhelm , A. J. Tavares , Q. Dai , et al., “Analysis of Nanoparticle Delivery to Tumours,” Nature Reviews Materials 1(2016): 16014.

[96] M. Soriano‐Garcia , “Organoselenium Compounds as Potential Therapeutic and Chemopreventive Agents: A Review,” Current Medicinal Chemistry 11(2004): 1657–1669. 15180570 10.2174/0929867043365053

[97] W. Yu , T. Liu , Z. Yuan , et al., “Polymer‐Engineered Liposome‐Delivered Ebselen against Tumor through GSH/H2O2‐Responsive Disruption of Redox Homeostasis and Direct p53 Activation,” Biomaterials 326(2026): 123698. 40992159 10.1016/j.biomaterials.2025.123698

[98] Y. Liang , B. Wang , Q. Yu , et al., “Ebselen Optimized the Therapeutic Effects of Silver Nanoparticles for Periodontal Treatment,” International Journal of Nanomedicine 18(2023): 8113–8130. 38169981 10.2147/IJN.S434579 PMC10759458

[99] F. Zhou , Y. He , M. Zhang , et al., “Polydopamine (PDA)‐Coated Diselenide‐Bridged Mesoporous Silica‐Based Nanoplatform for Neuroprotection by Reducing Oxidative Stress and Targeting Neuroinflammation in Intracerebral Hemorrhage,” Journal of Nanobiotechnology 22(2024): 731. 39578855 10.1186/s12951-024-03023-0 PMC11585243

[100] L. B. Macedo , D. R. Nogueira‐Librelotto , D. Mathes , et al., “Overcoming MDR by Associating Doxorubicin and pH‐Sensitive PLGA Nanoparticles Containing a Novel Organoselenium Compound—An In Vitro Study,” Pharmaceutics 14(2022): 80. 10.3390/pharmaceutics14010080 PMC8779681 35056975

[101] L. B. Macedo , D. R. Nogueira‐Librelotto , D. Mathes , et al., “Transferrin‐Decorated PLGA Nanoparticles Loaded with an Organoselenium Compound as an Innovative Approach to Sensitize MDR Tumor Cells: An In Vitro Study Using 2D and 3D Cell Models,” Nanomaterials 13(2023): 2306. 37630891 10.3390/nano13162306 PMC10458402

[102] S. Purohit , P. Oswal , A. Tyagi , et al., “Organoselenium Compounds as an Emerging Class of Stabilizers of Applied Nanomaterials for Applications in the Catalysis of Organic Reactions,” Asian Journal of Organic Chemistry 13(2024): e202400174.

[103] J. Yang , J. L. Welby , and M. E. Meyerhoff , “Generic Nitric Oxide (NO) Generating Surface by Immobilizing Organoselenium Species via Layer‐by‐Layer Assembly,” Langmuir 24(2008): 10265–10272. 18710268 10.1021/la801466e PMC2824255

[104] A. Arora , S. Singh , P. Oswal , et al., “Preformed Molecular Complexes of Metals with Organoselenium Ligands: Syntheses and Applications in Catalysis,” Coordination Chemistry Reviews 438(2021): 213885.

[105] A. Arora , P. Oswal , G. K. Rao , et al., “Organoselenium Ligands for Heterogeneous and Nanocatalytic Systems: Development and Applications,” Dalton Transactions 50(2021): 8628–8656. 33954317 10.1039/d1dt00082a

[106] A. Arora , P. Oswal , S. Singh , et al., “Organoselenium Ligand‐Stabilized Copper Nanoparticles: Development of a Magnetically Separable Catalytic System for Efficient, Room Temperature and Aqueous Phase Reduction of Nitroarenes,” Inorganica Chimica Acta 522(2021): 120267.

[107] P. Oswal , A. Arora , S. Gairola , et al., “Organosulfur, Organoselenium, and Organotellurium Ligands in the Development of Palladium, Nickel, and Copper‐Based Catalytic Systems for Heck Coupling,” New Journal of Chemistry 45(2021): 21449–21487.

[108] D. Sharma , A. Arora , P. Oswal , et al., “Organosulphur and Organoselenium Compounds as Emerging Building Blocks for Catalytic Systems for O‐Arylation of Phenols, a C–O Coupling Reaction,” Dalton Transactions 51(2022): 8103–8132. 35535745 10.1039/d1dt04371d

[109] K. Amporndanai , X. Meng , W. Shang , et al., “Inhibition Mechanism of SARS‐CoV‐2 Main Protease by Ebselen and Its Derivatives,” Nature Communications 12(2021): 3061. 10.1038/s41467-021-23313-7 PMC8144557 34031399

[110] N. A. Makhaeva , S. V. Amosova , A. S. Filippov , et al., “Recent Advances in Design, Synthesis, and Biological Activity Studies of 1,3‐Selenazoles,” Biomolecules 14(2024): 1546. 39766253 10.3390/biom14121546 PMC11674745

[111] P. Pyka , S. Garbo , R. Fioravanti , et al., “Selenium‐Containing Compounds: A New Hope for Innovative Treatments in Alzheimer's Disease and Parkinson's Disease,” Drug Discovery Today 29(2024): 104062. 38871111 10.1016/j.drudis.2024.104062

[112] K. I. Priyadarsini , B. G. Singh , P. P. Phadnis , K. C. Barick , and P. A. Hassan , “Nanoparticle conjugates of selenium compounds: Preparation, characterisation and electron transfer” in The 1st International Electronic Conference on Catalysis Sciences, (MDPI, 2020), 24.

[113] M. Kieliszek and S. N. Serrano Sandoval , “The Importance of Selenium in Food Enrichment Processes: A Comprehensive Review,” Journal of Trace Elements in Medicine and Biology 79(2023): 127260. 37421809 10.1016/j.jtemb.2023.127260

[114] J. Ma , C. Wang , Z. Wang , et al., “Active Dry Yeast Supplementation Improves the Growth Performance, Rumen Fermentation, and Immune Response of Weaned Beef Calves,” Animal Nutrition 7(2021): 1352–1359. 34786508 10.1016/j.aninu.2021.06.006 PMC8577086

[115] I. Di Leo , F. Messina , V. Nascimento , et al., “Synthetic Approaches to Organoselenium Derivatives with Antimicrobial and Anti‐Biofilm Activity,” Mini‐Reviews in Organic Chemistry 16(2019): 589–601.

[116] P. A. Tran and T. J. Webster , “Antimicrobial Selenium Nanoparticle Coatings on Polymeric Medical Devices,” Nanotechnology 24(2013): 155101. 23519147 10.1088/0957-4484/24/15/155101

[117] A. Bunker and T. Róg , “Mechanistic Understanding from Molecular Dynamics Simulation in Pharmaceutical Research: Drug Delivery,” Frontiers in Molecular Biosciences 7(2020): 604770. 33330633 10.3389/fmolb.2020.604770 PMC7732618

[118] I. D. Styliari , V. Taresco , A. Theophilus , C. Alexander , M. Garnett , and C. Laughton , “Nanoformulation‐by‐Design: An Experimental and Molecular Dynamics Study for Polymer Coated Drug Nanoparticles,” RSC Advances 10(2020): 19521–19533. 35515456 10.1039/d0ra00408a PMC9054057

[119] Y. Liu , X. Xiang , B. Wang , et al., “Maltotriose Acylhydrazone Surfactants: Structure–property Profiles and Efficient In Vivo Drug Delivery for Primary Biliary Cholangitis Treatment,” Journal of Molecular Liquids 390(2023): 123101.

[120] M. H. Mondal , S. Malik , A. Roy , R. Saha , and B. Saha , “Modernization of Surfactant Chemistry in the Age of Gemini and Biosurfactants: A Review,” RSC Advances 5(2015): 92707–92718.

[121] J. W. Agger and B. Zeuner , “Bio‐Based Surfactants: Enzymatic Functionalization and Production from Renewable Resources,” Current Opinion in Biotechnology 78(2022): 102842. 36371893 10.1016/j.copbio.2022.102842

[122] P. G. Argudo , L. Spitzer , E. Ibarboure , F. Jérôme , H. Cramail , and S. Lecommandoux , “Mannose‐Based Surfactant as Biofunctional Nanoemulsion Stabilizer,” Colloids and Surfaces B: Biointerfaces 220(2022): 112877. 36174495 10.1016/j.colsurfb.2022.112877

[123] T. Hellweg , T. Sottmann , and J. Oberdisse , “Recent Advances in Biosurfactant‐Based Association Colloids—Self‐Assembly in Water,” Frontiers in Soft Matter 2(2023): 1081877.

[124] E. Waglewska , A. Pucek‐Kaczmarek , and U. Bazylińska , “Novel Surface‐Modified Bilosomes as Functional and Biocompatible Nanocarriers of Hybrid Compounds,” Nanomaterials 10(2020): 2472. 33321762 10.3390/nano10122472 PMC7763575

